# Metabolic and Epigenetic Control of CD8^+^ T Cell Exhaustion: The Acetate‐to‐Citrate Switch

**DOI:** 10.1002/mco2.70307

**Published:** 2025-07-25

**Authors:** Xiaoyi Lei, Wei Zhang, Dunfang Zhang

**Affiliations:** ^1^ Department of Biotherapy State Key Laboratory of Biotherapy and Cancer Center Collaborative Innovation Center of Biotherapy West China Hospital Sichuan University Chengdu China; ^2^ Center for Immunology and Hematology State Key Laboratory of Biotherapy and Cancer Center West China Hospital Sichuan University Chengdu China

1

In a recent study published in *Science*, Ma et al. [1] identified a nutrient‐driven “acetate‐to‐citrate” metabolic switch that governs the epigenetic fate decisions of CD8^+^ T cells during chronic antigen stimulation. This study revealed that the subcellular localization and source‐specific generation of acetyl‐CoA modulate histone acetylation patterns, thereby directing T cells toward either an effector or exhausted phenotype. These findings provide a translational framework for enhancing T cell‐based therapies by metabolically reprogramming acetyl‐CoA availability.

CD8^+^ T cell exhaustion and dysfunction are critical contributors to the loss of effector function under chronic antigen exposure. During persistent stimulation, CD8^+^ T cells enter a progressively dysfunctional state known as terminal exhaustion (T_EX_), characterized by impaired effector function, sustained elevated expression of inhibitory receptors (e.g., PD‐1 and TIM‐3), transcriptional and epigenetic reprogramming, and significant metabolic alterations [2]. The epigenetic landscape of terminally exhausted T cells is stable, rendering their dysfunction difficult to reverse, even with immune checkpoint blockade (ICB) or chimeric antigen receptor T cell (CAR‐T) therapy, limiting therapeutic efficacy [3]. Understanding the epigenetic mechanisms driving CD8^+^ T cell differentiation and identifying key molecular “switches” that promote T_EX_ formation is, therefore, essential for optimizing T cell‐based immunotherapies and enhancing antitumor and antiviral responses.

Effector T cells (T_EFFs_) depend on glucose and amino acid metabolism for growth, proliferation, and function. In contrast, T_EX_ exhibits increased glycolysis, impaired mitochondrial function, and increased oxidized lipid uptake—metabolic shifts that compromise their antitumor capacity [4]. Emerging evidence suggests that such metabolic changes are not merely consequences but also active drivers of T cell fate. Notably, cellular metabolites directly influence epigenetic modifications. Acetyl‐CoA, a central metabolite, serves as an essential donor for histone acetylation and is a key epigenetic marker of active gene transcription. In mammalian cells, acetyl‐CoA is mainly generated through two enzymatic pathways: Acyl‐CoA synthetase short‐chain family member 2 (ACSS2), which converts acetate to acetyl‐CoA, and ATP citrate lyase (ACLY), which produces acetyl‐CoA from citrate [5]. However, the extent to which acetyl‐CoA levels, either globally or locally within specific subcellular compartments, regulate gene expression in CD8^+^ T cells remains unresolved.

Ma et al. [1] shed light on this fundamental question by demonstrating that nutrient availability and metabolic enzyme localization modulate histone acetylation patterns, determining CD8^+^ T cell fate. The researchers identified a previously unrecognized “acetate‐to‐citrate” metabolic switch during T_EX_ differentiation, driven by a shift from ACSS2 to ACLY dominance. Specifically, exhausted CD8^+^ T cells downregulate ACSS2 while maintaining ACLY expression, resulting in a transition in acetyl‐CoA production from acetate to citrate (Figure 1). This metabolic shift remodels the epigenetic landscape by differentially activating histone acetyltransferases (HATs). The ACSS2‐p300 complex promotes acetylation at effector and memory gene loci, supporting the T_EFF_ identity, while the ACLY‐KAT2A complex enhances acetylation at exhaustion‐associated loci, reinforcing the T_EX_ phenotype. This finding highlights that metabolic regulation directs the balance between effector function and exhaustion in T cells. The study reveals how the nutrient‐derived modifications of the “histone code” govern CD8^+^ T cell fate and offers new insight into metabolic reprogramming in chronic infections and cancer.

**FIGURE 1:**
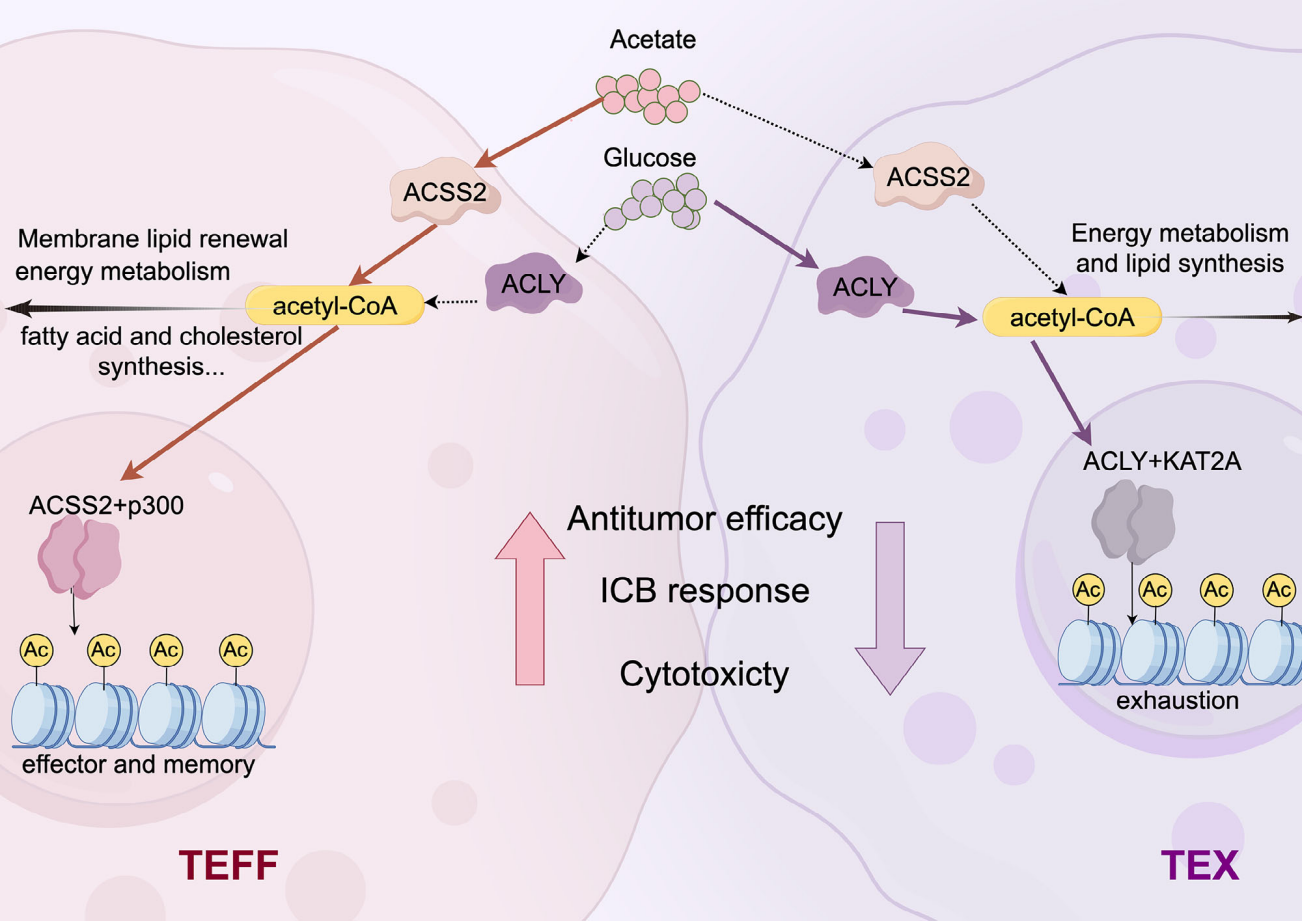
Metabolic Regulation of CD8^+^ T Cell Fate Decisions through Acetyl‐CoA and Histone Acetylation. This figure illustrates the distinct metabolic pathways and histone acetylation mechanisms that regulate effector T cell (T_EFF_) and terminal exhaustion (T_EX_) differentiation. In T_EFF_ cells (left), acetate‐derived acetyl‐CoA is produced by acyl‐CoA synthetase short‐chain family member 2 (ACSS2) and drives histone acetylation through the ACSS2‐p300 complex, promoting effector and memory gene expression and enhancing antitumor efficacy and cytotoxicity. In contrast, in T_EX_ cells (right), glucose‐derived acetyl‐CoA is produced by ATP citrate lyase (ACLY) and drives histone acetylation at exhaustion‐related gene loci via the ACLY‐KAT2A complex by downregulating ACSS2 expression. The shift in nutrient utilization—from acetate in T_EFF_ to glucose in T_EX_ cells—reflects a key metabolic and epigenetic switch that drives T cell differentiation and contributes to immune dysfunction in chronic infections and cancer. This mechanism highlights the potential for targeting metabolic pathways to enhance immune responses in cancer therapy. ICB: immune checkpoint blockade. Figure 1 was created with FigDraw.

Further studies demonstrated that the subcellular localization of acetyl‐CoA‐producing enzymes plays a decisive role in regulating histone acetylation. Localized acetyl‐CoA pools determine HAT activity and selectively modulate gene expression. HAT activity ultimately determines T cell fate, either by maintaining T_EFF_ function or promoting T_EX_ differentiation. Consequently, metabolic regulation plays a pivotal role in shaping the functional outcomes of T cells during the immune response. These findings provide mechanistic insights into the localization of metabolite pools that selectively influence chromatin states and gene expression, highlighting a critical axis connecting metabolism, epigenetics, and T‐cell function.

To explore translational applications, the researchers overexpressed nuclear ACSS2 and inhibited ACLY in experimental models. Furthermore, ACLY targeting in human CAR‐T cells restored effector functions and improved antitumor efficacy, suggesting that modulating acetyl‐CoA metabolism represents a promising strategy for enhancing the performance of T cell‐based immunotherapies through targeted metabolic reprogramming.

In conclusion, this study presents a compelling framework for the metabolic‐epigenetic crosstalk underlying T‐cell exhaustion. In addition to serving as a metabolic intermediate, nutrient‐derived acetyl‐CoA is highlighted as a spatially regulated epigenetic modulator. By shifting the focus from global metabolite levels to localized metabolic signaling, the study provides a nuanced understanding of localized metabolic control. This concept underscores the importance of spatially regulated metabolic signals in defining T‐cell function and differentiation. By integrating metabolism and epigenetics, this discovery provides a theoretical foundation for developing novel immunotherapies, particularly those aimed at enhancing immune responses by T cell metabolic reprogramming.

## Author Contributions

**X.L**. drafted the manuscript. **W.Z**. edited the manuscript. **D.Z**. supervised the work and edited the manuscript. All authors have read and approved the final manuscript.

## Ethics Statement

Not Applicable.

## Conflicts of Interest

The authors declare no conflict of interest.

## Data Availability

Not Applicable.
